# 429. Identifying Barriers to Compliance with a Universal Inpatient Protocol for *Staphylococcus aureus* Nasal Decolonization with Povidone-Iodine

**DOI:** 10.1093/ofid/ofac492.504

**Published:** 2022-12-15

**Authors:** Thomas R Talbot, Bryan D Harris, Mary DeVault, Rebecca A Stern

**Affiliations:** Vanderbilt University Medical Center, Nashville, Tennessee; Vanderbilt University Medical Center, Nashville, Tennessee; Vanderbilt University Medical Center, Nashville, Tennessee; Vanderbilt University Medical Center, Nashville, Tennessee

## Abstract

**Background:**

Intranasal povidone-iodine (PI) is a recommended strategy for universal decolonization in high-risk patients (ICU and those with central venous or midline catheters) to reduce hospital-associated Staphylococcal infections. Few studies have evaluated implementation challenges and barriers to successful performance of inpatient intranasal decolonization programs.

**Methods:**

We surveyed adult acute care unit nurses at an academic medical center in March 2022, approximately 14 months after implementation of a universal decolonization standard operating procedure (SOP). The anonymous, voluntary REDCap® survey evaluated domains focused on patient identification, education, training, resources, application, and patient acceptance using Likert scale ratings.

**Results:**

Among 248 respondents, most were new to nursing (54.4% with 0-4 years of experience) and worked in non-ICU units (61.5%). Only 60.5% reported receiving training on how to perform intranasal PI (hands-on 48.6%, computer/electronic module 25.7%, both 20.9%). Nurses who received training indicated moderate to strong confidence in their ability to perform intranasal PI decolonization (89.2%). A majority cited a good understanding of the rationale for use and identified patients appropriately. Low rates were reported for performing decolonization per the SOP (49%), with barriers including inadequate supplies (35.1%), lack of a readily available copy of the SOP (69%), difficulty swabbing with nasal devices in place (41.5%) and time constraints from other patient duties. Nurses perceived that only 49.2% of patients had a moderate or strong understanding of why PI decolonization was performed, and most were unwilling to undergo intranasal PI (59.1%). Other issues included tracking PI application within the electronic medical record (EMR), limited nurse knowledge of PI effectiveness, patient refusal despite education, and overall frontline personnel burnout.

**Figure:**
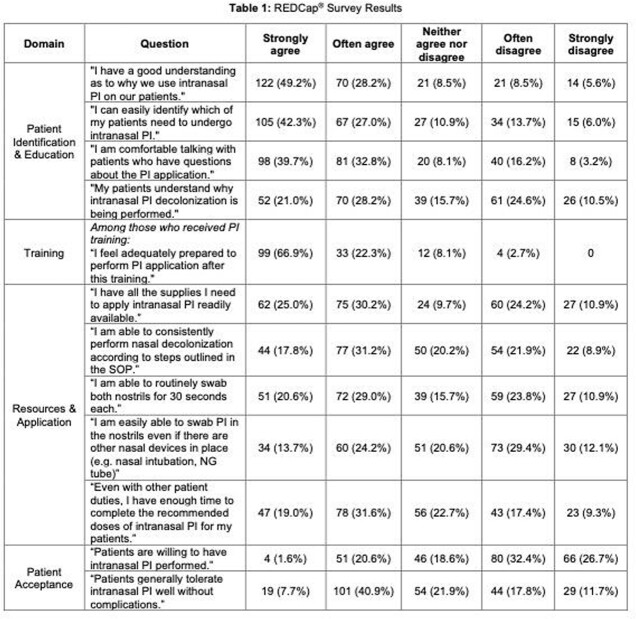

**Conclusion:**

Gaps in nursing and patient education should be prioritized during and after implementation to improve fidelity, particularly with frontline burnout from COVID-19. Streamlined tracking and ordering of PI on the EMR may ease nursing workflow.

**Disclosures:**

**Thomas R. Talbot, III, MD, MPH**, OmniSolve: Board Member.

